# Unbiased and Automated Identification of a Circulating Tumour Cell Definition That Associates with Overall Survival

**DOI:** 10.1371/journal.pone.0027419

**Published:** 2011-11-07

**Authors:** Sjoerd T. Ligthart, Frank A. W. Coumans, Gerhardt Attard, Amy Mulick Cassidy, Johann S. de Bono, Leon W. M. M. Terstappen

**Affiliations:** 1 Medical Cell BioPhysics group, MIRA Institute, University of Twente, Enschede, The Netherlands; 2 The Royal Marsden National Health Service Foundation Trust, Sutton, Surrey, United Kingdom; National Cancer Center, Japan

## Abstract

Circulating tumour cells (CTC) in patients with metastatic carcinomas are associated with poor survival and can be used to guide therapy. Classification of CTC however remains subjective, as they are morphologically heterogeneous. We acquired digital images, using the CellSearch™ system, from blood of 185 castration resistant prostate cancer (CRPC) patients and 68 healthy subjects to define CTC by computer algorithms. Patient survival data was used as the training parameter for the computer to define CTC. The computer-generated CTC definition was validated on a separate CRPC dataset comprising 100 patients. The optimal definition of the computer defined CTC (aCTC) was stricter as compared to the manual CellSearch CTC (mCTC) definition and as a consequence aCTC were less frequent. The computer-generated CTC definition resulted in hazard ratios (HRs) of 2.8 for baseline and 3.9 for follow-up samples, which is comparable to the mCTC definition (baseline HR 2.9, follow-up HR 4.5). Validation resulted in HRs at baseline/follow-up of 3.9/5.4 for computer and 4.8/5.8 for manual definitions. In conclusion, we have defined and validated CTC by clinical outcome using a perfectly reproducing automated algorithm.

## Introduction

In recent years, several studies have reported that a change in circulating tumour cell (CTC) count could indicate whether a therapy for advanced cancer is effective [1], [2], [3], [4], [5], [6], [7]. It is envisioned that the clinical use of CTC as a pharmacodynamic and predictive biomarker will rapidly increase in the near future, especially in advanced prostate and breast cancers [8]. Currently, the CellSearch™ method is the only clinically validated and FDA-cleared method for CTC enumeration [9]. In this system, objects that are positive for epithelial cell adhesion molecule (EpCAM) antigen are enriched from 7.5 ml of blood and then stained with cytokeratin-phycoerythrin (CK-PE), CD45-allophycocyanin (CD45-APC) and the nuclear dye 4*#*,6-diamidino-2-phenylindole (DAPI). The recorded fluorescence images of CK-PE, DNA-DAPI, CD45-APC and a debris-fluorescein (FITC) channel are segmented on the basis of being positive for CK-PE and DAPI and are then presented to a trained reviewer for identification of CTC that are CK-PE positive, CD45-APC negative, ≥4 µm in diameter, DAPI-positive, and have a cell-like morphology. This manual procedure is laborious, time-consuming and can be highly subjective. For example, others have described an inter-reviewer variation in manual CTC enumeration of 4% to 31% (median 14%) [10]. Moreover, CTC are known to be morphologically heterogeneous and in fact, different laboratories have used different definitions for what constitutes a CTC, especially for objects that are dead or apoptotic [2], [10]. CTC can occur at very low frequencies and therefore misjudging a few events could be very significant [11]. Also, the definition of what to call a CTC that is currently used may not be optimal. A recent report showed that tumour micro particles (TMPs) -EpCAM+CK+CD45- objects smaller than 4 µm- are also associated with poor prognosis, suggesting alternative definitions for CTC evaluation should be considered [12].

Here we present the results of a new approach to identify CTC in images captured by the system in samples from castration-resistant prostate cancer (CRPC) patients. We recorded images before treatment (baseline samples) and from a follow-up sample taken 2–6 weeks after start of therapy. Our hypothesis was that using survival data as the only training parameter, an automated algorithm could be optimized to define and automatically count CTC with the same fidelity as the manual CellSearch method (mCTC). This algorithm needs to identify automated CTC (aCTC) candidates, characterize them and compare the candidates to a range of known features. Replacement of manual CTC counting with an automated method would significantly reduce cost and importantly, eliminate inter- and intra-laboratory variation that could be clinically important in cases with low CTC counts. Moreover, a consensus definition for what constitutes a CTC is urgently required [13]. By using an unbiased approach to identify clinically important events, our analyses informs on the validity of different criteria currently being used, which were validated on an independent data set.

## Methods

### Ethics Statement

Development of image analysis algorithms for automated CTC enumeration was performed on stored images from ten CellSearch systems (Veridex LLC, Raritan, NJ) from patients participating in the prospective IMMC-38 study (NCT00133900) and healthy individuals participating in the IMMC-06 study (NCT00133913) were available [7], [14]. For validation of the algorithm, images were used from samples from patients participating in Phase I and II clinical studies of abiraterone acetate (NCT00473512) conducted at the Royal Marsden NHS Foundation Trust and reported previously [15], [16], [17]. Samples were processed at The Institute of Cancer Research (ICR) (Sutton, UK) and archived images were sent for automated analysis at the University of Twente (Netherlands). The University of Twente was blinded to survival data for the validation samples. These studies were approved by the Ethics Review Committees of the participating centres: the United States Institutional Review Board for IMMC-38; the United States Food and Drug Administration and the United Kingdom Medicines and Healthcare Products Regulatory Agency for abiraterone acetate. All patients and healthy individuals provided written informed consent.

### Participants

All patients had histologically confirmed prostate adenocarcinoma, castrate levels of testosterone (<50 ng/ml) and progressive disease as defined by three consecutively rising PSA values [18]. Patients included in IMMC-38 were commencing a new cytotoxic therapy. Patients with brain metastases or a history of other malignancies within the last 5 years were excluded. 276 patients were enrolled in IMMC-38, 231 met eligibility criteria and for 185 of those patients images could be imported for baseline and first follow-up [14]. Baseline samples were taken up to 19 days prior to commencement of a new cytotoxic chemotherapy, follow-up samples were taken 2–6 weeks after the start of therapy. 121 patients started their first line of chemotherapy. A total of 65 clinical centres in the United States and Europe participated in this study. In the abiraterone acetate studies, samples were collected from a total of 100 patients. 89 patients contributed both a baseline and a follow-up sample, 7 contributed only follow-up, 4 only baseline. Fifty-one patients were chemotherapy naïve, and 44 patients were docetaxel-pretreated. Samples collected up to 14 days before initiation of abiraterone acetate (93 samples) and after one cycle (28 days) of therapy (96 samples) were used for this analysis. Of 185 IMMC-38 patients 118 (64%) died, in the abiraterone acetate studies 73 of 100 (73%) died. Median survival was 20.7 months for IMMC-38 and 31.5 months for abiraterone acetate. Median duration of follow-up for censored patients was 29.8 months for IMMC-38 and 41.8 months for abiraterone acetate. In addition, samples of 68 healthy individuals participating in the IMMC-06 study were available [7]. Healthy individuals donated blood at three clinical centres in the US, the Netherlands, and the United Kingdom.

### Manual Counting of Circulating Tumour Cells (mCTC)

The CellSearch system was used to isolate and image EpCAM+ objects. The CellSearch system consists of a CellTracks Autoprep for sample preparation [2], [9] and a CellTracks Analyzer II for sample analysis. The CellTracks Autoprep immuno-magnetically enriches epithelial cells from 7.5 ml of blood using ferrofluids conjugated to epithelial cell adhesion molecule antibodies (EpCAM). The enriched sample is stained with phycoerythrin-conjugated (PE) antibodies directed against cytokeratins 8, 18, and 19 (CK), an allophycocyanin-conjugated (APC) antibody to CD45 and the nuclear dye 4*#*,6-diamidino-2-phenylindole (DAPI). This enriched sample is transferred to a magnetic cartridge where all ferrofluid labeled objects are pulled towards an analysis surface. The entire analysis surface is imaged by the CellTracks Analyzer II, a four-color semi-automated fluorescence microscope that captures digital images for four different fluorescent dyes using a 10X/0.45NA objective. In addition to the DAPI, PE and APC images, a fourth fluorescence channel (emission 535±25 nm) is imaged as a control channel for exclusion of auto-fluorescent debris. This channel will be termed “FITC” channel. Per cartridge, a whole scan consists of 144–180 4-layer tiff images that are saved for each patient. After imaging by the Celltracks analyzer, the software selects objects that are DNA and CK positive and presents them to an operator in a thumbnail gallery. The operators are trained to reviews these galleries to select the mCTC among the objects. An mCTC is positive for DNA and CK, is negative for CD45, is larger than 4×4 µm and has morphological features that are consistent with those of a cell.

### Automated counting of EpCAM+ objects using a computer algorithm (aCTC)

**Figure 1:**
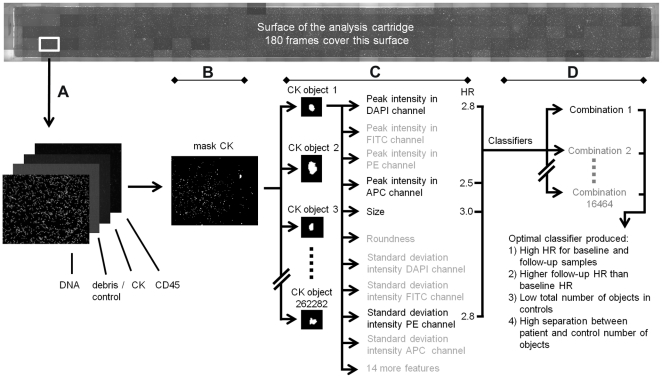
Schematic overview of the aCTC classifier development process. A: importing of images; B: object segmentation; C: feature measurements; D: classification of aCTC.

CDs containing up to 180 archived four channel tiff images for each sample belonging to the respective studies were collected for import to a central hard drive. Objects were detected and classified using an automated algorithm developed in Matlab 2009a (Mathworks, Natick, MA) using the DIPimage toolbox (www.diplib.org). An outline of the method is given below and shown in figure 1. The method was applied for each patient sample separately. First, the true imaging area where all the objects were located was determined via sample cartridge edge detection in the debris-FITC channel. Candidate CTC objects were selected via object segmentation in the CK-PE channel. Segmentation was performed using a threshold which was determined for each sample via the channel image histogram [19]. Applying this threshold to the CK-PE images returned the outline, size and location of the objects. In the next step, measurements, termed features from here on, were performed on these objects and the features providing the largest Cox hazard ratio (HR, shown next to the features in figure 1) and low correlation with other selected features were chosen for classification of these objects: the standard deviation of the signal in the CK-PE channel, the peak signal value in both the DNA-DAPI and CD45-APC channels and the size of the objects. Finally, selection of aCTC was performed by comparing every object to numerical inclusion criteria for these four features. The combined inclusion criteria -termed classifier from here on- were varied to find the aCTC definition that most strongly associated with high HR for baseline and follow-up samples, a higher HR for follow-up than baseline samples, and a low relative and absolute count in control samples. Bootstrap aggregation was used to test the stability of the optimal classifier [20].

### Statistical analysis

The primary objective was to identify a CTC definition with the largest HR between favourable and unfavourable patient groups and a low background in the control group. During algorithm development, the median number of events found was used to dichotomize patients into two groups. This approach allowed quick selection of a threshold, while ensuring that sufficient patients are present in both the ‘at risk’ and the ‘not at risk’ groups. It also allowed comparison of HR determined for different features and minimized error in HR. For this approach to work there needs to be a continuous relationship between survival and CTC count, which was previously demonstrated for the IMMC-38 data [21].

After algorithm optimization, all patient samples were processed by the algorithm and the training and validation patient groups were dichotomized on cut-off values ranging 1–10 of aCTC and mCTC to derive HR and median overall survival (OS) for baseline and first follow-up samples. Furthermore, a linear regression was performed for comparison between the aCTC and mCTC count. Classifiers with reduced features were tested to determine the impact of each feature. Pearson coefficient of determination R^2^ was determined between these populations using Matlab. Statistical HR and Kaplan Meier analysis for the training dataset was performed by S.T.L. using Matlab and GraphPad Prism v5. Statistical HR and Kaplan Meier analysis for the validation dataset was performed by A.M.C. at the ICR using Stata v10.1 (StataCorp) and GraphPad Prism v5.

## Results

### Choosing the optimal classifier and processing of samples

The classifier resulting in the optimal aCTC definition that most strongly associated with high HR for baseline and follow-up samples was chosen. The features that most strongly associated with OS were: a CK-PE standard deviation >50 counts, a size range of 75–500 pixels (34–224 µm^2^), a DAPI-DNA peak value >170 counts and a CD45-APC peak value <60 counts. For every patient sample, the objects meeting these inclusion criteria were added up to arrive at a final aCTC count per patient. The mCTC count was performed by trained reviewers. Time needed for preparation of images for mCTC assignment was similar to the time needed for complete aCTC enumeration; both took 5 minutes. However, enumeration of these mCTC by a human operator takes an additional 8 minutes per sample (median 5, range 1–39, SD 8 minutes, N = 43).

### Automated CTC count compared to manual CTC count in patients and controls

**Figure 2:**
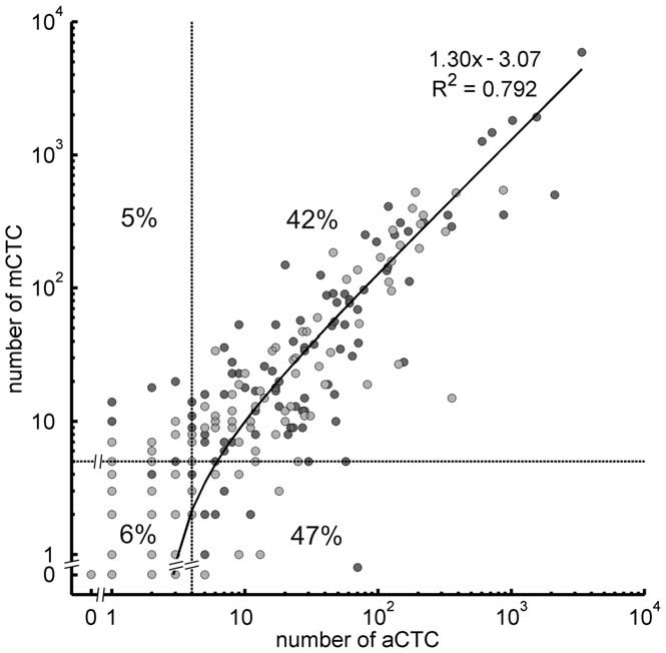
Scatter plot of baseline (dark grey) and first follow-up (light grey) samples, counted by the aCTC and mCTC methods. The linear regression statistics apply to the total data set. Quadrants were defined by the clinically used cut-off value of 5 mCTC and the empirical determined value of 4 aCTC (dashed lines). In each quadrant, the percentage of patients is shown.

After all the objects meeting the criteria of the optimal classifier were summed for each patient sample, the aCTC count was compared with the mCTC count. In the baseline samples the aCTC counts ranged from 0 to 3384 (median 5, mean 78, SD 333) compared to mCTC counts of 0 to 5925 (median 7, mean 101, SD 497). The R^2^ between aCTC and mCTC was 0.80 (slope = 1.33, intercept = −3.03). In the follow-up samples aCTC counts ranged from 0 to 870 (median 2, mean 27, SD 86) compared to mCTC counts of 0 to 545 (median 2, mean 30, SD 87). The R^2^ was 0.67 (slope = 0.85, intercept = 7.18). Figure 2 shows a scatter plot of the baseline and follow-up samples with the linear regression and corresponding statistics of the combined baseline and follow-up samples. In 68 control samples only one object was classified as aCTC and zero objects as mCTC.

**Figure 3:**
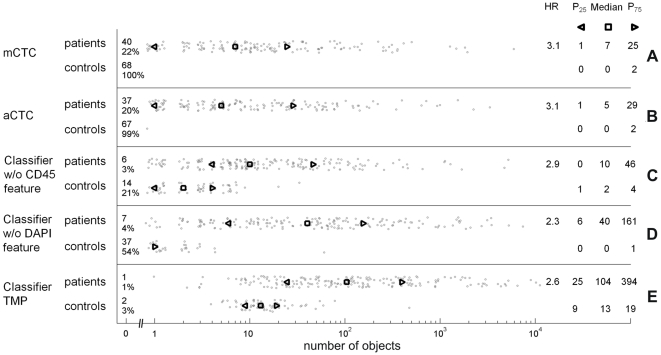
Frequency distributions of mCTC and aCTC from patients (N = 185) and control samples (N = 68). The top row shows the mCTC frequency distribution (panel A). The next rows show the number of aCTC for the optimal aCTC definition (panel B), the classifier without the CD45 exclusion criterion (panel C), without the DAPI criterion (panel D), and of TMP objects that are EpCAM+CK+CD45- and <4 µm in diameter (panel E). The percentage of patients with 0 objects is shown numerically on the left. On the right the HRs derived by dichotomizing on the median number of objects in patients are shown, together with the 25, 50, and 75 percentiles. The percentiles are also indicated in the plot.

Figure 3 shows the frequency distributions of mCTC (median 7) and aCTC (median 5) in baseline samples from patients and in controls for the optimal aCTC definition and three other definitions that are less strict: without the CD45 feature (median 10), without the DAPI feature (median 40), and for TMP objects that are EpCAM+CK+CD45- (CK standard value >10 counts; CD45 peak value <60 counts ) and <4 µm in diameter (median 104). R^2^ between aCTC and mCTC was 0.78. Between aCTC and the objects found with the classifiers without CD45 and DAPI the R^2^ were 0.95 and 0.82, respectively. Between the aCTC and TMP definition, the R^2^ was 0.56 (p<0.0001 for all R^2^).

### Defining cut-off values for aCTC and mCTC

To arrive at a clinically relevant cut-off value for aCTC comparable to the mCTC cut-off of 5 used in routine clinical practice, we used the linear regression slope of 1.33 between aCTC and mCTC baseline measurements. This resulted in a cut-off of 4 aCTC. In the scatter plot of figure 2 the cut-offs are indicated creating four quadrants: two with concordant and two with discordant results. The percentage of patients in each quadrant is provided. A total of 11% of patients had discordant results based on the CTC cut-offs of mCTC and aCTC. The influence of other CTC cut-off values from ≥1 to ≥10 CTC on the number of patients affected, the median OS, HR and its significance for both baseline and follow-up samples were determined and are shown in table 1 (p-values for all HRs<0.0001, except baseline cut-off = 1: p = 0.0003 for aCTC and p = 0.004 for mCTC).

**Table 1:** aCTC and mCTC cut-off values with HRs, median OS, for baseline (panel A) and first follow-up samples (panel B) for the training set enrolled in the IMMC-38 study.

A (N = 185)	Classifier				CellSearch			
cut off	n≥cut off (%)	50% OS*<cut off	50% OS≥cut off	HR^†^ (95% CI)	n≥cut off (%)	50% OS<cut off	50% OS≥cut off	HR (95% CI)
1	147 (79)	33.0	17.5	2.6 (1.6–4.5)	145 (78)	32.0	17.4	2.0 (1.2–3.2)
2	126 (68)	33.3	15.8	3.0 (1.9–4.8)	128 (69)	33.3	15.6	2.8 (1.8–4.4)
3	115 (62)	33.3	15.2	3.0 (1.9–4.6)	118 (64)	33.1	15.2	2.9 (1.9–4.4)
4	105 (57)	33.1	14.5	2.8 (1.9–4.1)	108 (58)	32.1	14.5	2.5 (1.7–3.7)
5	93 (50)	31.2	13.6	3.1 (2.1–4.7)	104 (56)	32.0	14.5	2.9 (2.0–4.4)
6	87 (47)	32.0	12.5	3.0 (2.0–4.5)	96 (52)	32.0	13.6	2.8 (1.9–4.2)
7	82 (44)	31.0	11.5	3.2 (2.2–4.8)	95 (51)	32.0	13.6	3.1 (2.1–4.5)
8	75 (41)	30.6	11.4	3.1 (2.1–4.6)	87 (47)	31.2	11.5	3.2 (2.1–4.7)
9	72 (39)	30.6	11.4	3.0 (2.0–4.4)	84 (45)	31.2	11.4	3.4 (2.3–5.0)
10	70 (38)	29.0	11.4	2.9 (1.9–4.3)	78 (42)	30.6	10.6	3.2 (2.1–4.7)
**B** (N = 185)								
1	129 (70)	31.4	15.2	2.3 (1.6–3.5)	110 (59)	33.4	12.5	3.0 (2.1–4.5)
2	98 (53)	29.6	11.6	3.6 (2.4–5.3)	94 (51)	31.1	11.5	3.9 (2.6–5.7)
3	79 (43)	29.6	9.8	4.0 (2.7–5.9)	81 (44)	31.2	10.3	4.4 (2.9–6.5)
4	72 (39)	29.6	9.6	3.9 (2.6–5.9)	77 (42)	31.2	10.3	3.8 (2.5–5.6)
5	67 (36)	28.3	9.5	4.8 (3.2–7.3)	71 (38)	30.6	10.5	4.5 (3.0–6.8)
6	62 (34)	28.3	9.4	4.4 (2.9–6.6)	64 (35)	29.3	10.2	4.7 (3.1–7.1)
7	57 (31)	25.7	9.4	4.4 (2.9–6.6)	62 (34)	29.3	10.2	4.5 (3.0–6.8)
8	57 (31)	25.7	9.4	4.5 (2.9–6.8)	58 (31)	28.2	9.2	4.7 (3.1–7.1)
9	53 (29)	25.7	9.4	4.0 (2.6–6.1)	55 (30)	28.2	9.2	4.7 (3.1–7.2)
10	49 (26)	25.6	9.4	3.9 (2.6–6.0)	51 (28)	27.5	9.2	4.3 (2.8–6.6)

*Overall Survival; ^†^Hazard Ratio.

**Figure 4:**
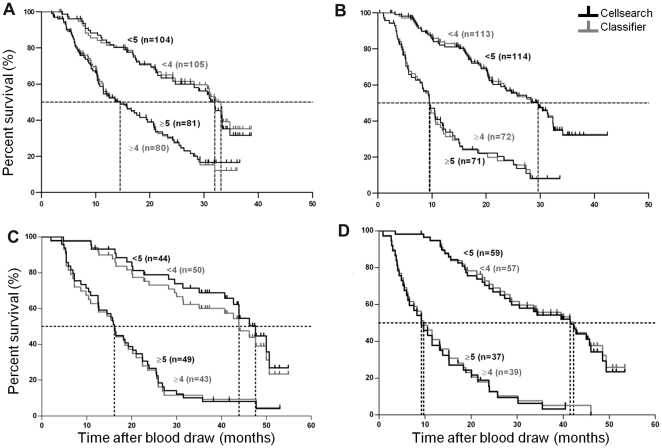
Kaplan-Meier plots of the classifier (grey lines) and the manual CellSearch (black lines) definition. The training set is shown in panel A (baseline, N = 185) and panel B (follow-up, N = 185). Kaplan-Meier plots for the validation set are shown in panel C (baseline, N = 93) and panel D (follow-up, N = 96). Censoring is indicated by vertical marks on the Kaplan-Meier plot.

Kaplan-Meier plots were generated for 185 baseline and 185 follow-up samples using the standard cut-off value of 5 for mCTC and the cut-off value for aCTC of 4. Figure 4, panel A shows the Kaplan-Meier plot for the baseline samples. Cox regression yielded a HR of 2.8 (95% CI 1.9–4.1) for aCTC and a HR of 2.9 (95% CI 2.0–4.4) for mCTC. Figure 4, panel B shows the Kaplan-Meier plot for the follow-up samples. For the first follow-up samples we found a HR of 3.9 (95% CI 2.6–5.9) for aCTC and a HR of 4.5 (95% CI 3.0–6.8) for mCTC.

### Validation of automated CTC count

To validate the aCTC count an independent data set was used from 100 metastatic prostate cancer patients treated with abiraterone acetate. The set included 93 baseline samples 96 follow-up samples. In the baseline samples the aCTC counts range was 0–1258 (median 3, mean 46, SD 152) and a range of 0–1108 (median 6, mean 53 SD 151) was found for mCTC. R^2^ between aCTC and mCTC was 0.28 (slope = 0.52, intercept = 28.76). Exclusion of a single outlier resulted in a R^2^ of 0.90 (slope 1.72, intercept 4.07). In the follow-up samples the aCTC counts range was 0–2490 (median 2, mean 78, SD 326) and a range of 0–3573 (median 2, mean 74, SD 390) with mCTC. R^2^ between aCTC and mCTC both was 0.83 (slope = 1.09, intercept = −11.43).

Kaplan-Meier plots were generated for 93 baseline and 96 follow-up samples using the standard CTC cut-off of 5 for mCTC and 4 for aCTC. Figure 4 Panel C shows the Kaplan-Meier plot from the baseline samples. Cox regression resulted in a HR of 3.9 (95% CI 2.4–6.6) for aCTC and a HR of 4.8 (95% CI 2.8–8.3) for mCTC. The Kaplan Meier plot from follow-up samples is presented in figure 4, panel D. A HR of 5.4 (95% CI 3.2–8.9) was found for aCTC and a HR of 5.8 (95% CI 3.4–9.8) for mCTC (p-values for all HRs<0.0001). Table 2 shows the influence of other cut-off values on the HR and OS.

**Table 2:** aCTC and mCTC cut-off values with HRs, median OS, for baseline (panel A) and first follow-up samples (panel B) for the validation set enrolled in Phase I/II trials of abiraterone acetate at Royal Marsden Hospital.

A (N = 93)	Classifier				CellSearch			
cut off	n≥cut off (%)	50% OS*<cut off	50% OS≥cut off	HR† (95% CI)	n≥cut off (%)	50% OS<cut off	50% OS≥cut off	HR (95% CI)
1	69 (74)	50.0	20.3	4.3 (2.2–8.7)	60 (65)	50.0	18.5	3.8 (2.1–6.9)
2	54 (58)	46.2	18.1	3.3 (2.0–5.7)	56 (60)	47.6	18.1	4.0 (2.3–7.0)
3	49 (53)	46.2	16.3	4.0 (2.4–6.8)	50 (54)	47.6	16.1	4.8 (2.8–8.4)
4	43 (46)	44.0	16.1	3.9 (2.4–6.6)	49 (53)	47.6	16.1	4.8 (2.8–8.3)
5	43 (46)	44.0	16.1	3.9 (2.8–6.6)	49 (53)	47.6	16.1	4.8 (2.8–8.3)
6	41 (44)	43.9	15.9	3.8 (2.3–6.2)	47 (51)	46.2	16.1	4.4 (2.6–7.4)
7	37 (40)	43.9	15.4	3.6 (2.2–5.9)	45 (48)	44.0	16.3	3.9 (2.3–6.5)
8	36 (39)	42.7	14.5	3.5 (2.2–5.8)	43 (46)	44.0	16.1	3.8 (2.3–6.4)
9	35 (38)	42.7	15.4	3.3 (2.0–5.4)	41 (44)	43.9	15.9	3.6 (2.2–5.9)
10	33 (35)	40.7	15.4	3.0 (1.8–4.9)	40 (43)	43.9	15.7	3.5 (2.1–5.7)
**B** (N = 96)								
1	58 (60)	48.5	13.8	5.4 (3.0–9.9)	55 (57)	48.5	13.8	5.4 (3.1–9.6)
2	51 (53)	45.0	13.3	5.0 (2.9–8.6)	50 (52)	45.0	11.5	5.0 (2.9–8.5)
3	40 (42)	42.2	9.7	5.6 (3.4–9.4)	44 (46)	42.2	11.1	4.8 (2.9–8.0)
4	39 (41)	42.2	9.7	5.4 (3.2–8.9)	40 (42)	42.2	9.7	6.6 (3.8–11.4)
5	37 (39)	41.5	9.7	4.9 (3.0–8.1)	37 (39)	41.5	9.2	5.8 (3.4–9.8)
6	34 (35)	41.5	9.2	6.8 (3.9–11.6)	36 (38)	41.5	9.2	6.2 (3.7–10.5)
7	33 (34)	41.5	9.2	6.6 (3.8–11.1)	36 (38)	41.5	9.2	6.2 (3.7–10.5)
8	32 (33)	41.5	9.2	6.1 (3.6–10.4)	35 (36)	41.5	9.2	5.9 (3.5–10.0)
9	30 (31)	39.5	9.2	6.2 (3.6–10.5)	32 (33)	39.5	7.6	5.3 (3.2–8.9)
10	28 (29)	34.4	9.2	5.5 (3.3–9.5)	32 (33)	39.5	7.6	5.3 (3.2–8.9)

*Overall Survival.

† Hazard Ratio.

## Discussion

This is the first report of an algorithm-based automated method for unbiased determination of a clinically significant definition for what constitutes a CTC. We used stored images recorded by the CellSearch system from 185 patients with metastatic CRPC. While the algorithm was developed using patients with metastatic CRPC receiving a cytotoxic agent (training cohort), validation on patients receiving the highly active hormonal agent abiraterone acetate (validation cohort) confirmed reproducibility of the enumeration algorithm.

For the purpose of the development of the CTC classifier, patients were divided into two groups based on the median aCTC count in the training set of 185 baseline samples. This division was chosen to minimize statistical error in the HR. The median count for the chosen aCTC classifier on the baseline samples was 5 aCTC and resulted in a HR of 3.1. The current standard CellSearch method presents the reviewer with CK+ DAPI+ objects for classification: a threshold of 5 or more mCTC is used to discriminate between patients with a favourable versus an unfavourable prognosis. In an earlier study we reported that this threshold of 5 mCTC could be mainly attributed to error introduced by human interpretation [11]. The variability of counting aCTC by the algorithm is 0% compared to inter-reviewer variability of 4% to 31% for mCTC (median 14%) [10]. As we have eliminated this variability by using an automated method, one could argue that the presence of any CTC could now be used to identify patients at risk. To identify a threshold for aCTC we used the correlation statistics between mCTC and aCTC and proposed a cut-off for aCTC of 4. As shown in figure 4, the Kaplan-Meier plots before and after one cycle of treatment using aCTC and mCTC are equivalent. The aCTC classifier that was chosen detected up to one object in the control samples as can be seen in figure 3. From this figure it becomes clear that a CD45 exclusion criterion is necessary to suppress the number of background objects, although the impact on the HR is small. The influence of the DAPI exclusion criterion is large on HR: only cells with sufficient DNA should be included.

Although one would expect that counting tumour related events that occur at a higher frequency -such as TMPs [12]- is more sensitive and robust, the relationship with clinical outcome was less strong. The aCTC definition was stricter as compared to the mCTC definition as is exemplified by the frequency differences. R^2^ between TMP and aCTC was 0.56 and TMPs were also present in the control group (see figure 3 bottom row). This may suggest that the current definition of TMPs is a proxy for the number of viable CTC, but in addition enumerates objects unrelated to tumor metastasis (e.g. originating from cell death in tumor or healthy tissue). Higher numbers of events are needed to improve robustness. TMPs may provide these higher numbers, but additional markers are needed to suppress the background signal in healthy volunteers.

The aCTC definition was validated using an independent data set. This validation set showed that the classifier performs well with equivalent HRs to those obtained with mCTC. Correlation with mCTC was quite low (R^2^ 0.28) due to one outlier. For this outlier, the algorithm counted 1258 aCTC, whereas the operator only counted 67 mCTC. Closer inspection of this sample revealed that this sample had a very high density of cells. This resulted in an overestimation of the number of CTC by the algorithm and an underestimation of the number of CTC by the human operator. Kaplan-Meier plots of baseline and follow-up using aCTC and mCTC from the validation set illustrated in figure 4 strongly support the use of the aCTC for routine clinical use. Whether or not the same definition for an aCTC can be used for other cancers remains to be determined and is currently being investigated in a large number of samples from breast and colorectal cancer patients. The definition of aCTC in this study was optimized towards the clinical outcome of the patients and developed using stored images taken with a 10X/0.45NA objective. The imaged objects were selected immune-magnetically targeting the EpCAM antigen and stained with DAPI, CD45-APC, Cytokeratin 8,18 & 19-PE. Alteration of the microscope or reagents used to identify the CTC will obliterate the aCTC definition. To use this approach for other CTC capturing methods clinical studies will need to be conducted and images stored for relating the particular CTC definition to clinical outcome.

The CellSearch system is the first and currently the only clinically validated method for CTC enumeration. The system was introduced in 2004 and its initial users were well-trained clinical researchers. The need for CTC counts in the clinic to manage patients with metastatic disease is however rising quickly and is accompanied by a need for simplification, higher reproducibility and a reduction of time needed to obtain a result, i.e. cost reduction. The introduction of aCTC addresses these issues as the need for extensively trained reviewers is eliminated, the algorithm is perfectly reproducible and no operator time is needed to review the images.

In conclusion, we have identified and validated a definition for CTC using an unbiased, automated algorithm that confirms that CK+DAPI+CD45- cells are the EpCAM positive events most strongly associated with survival. Moreover, automated counting of CTC using our classifier compares favourably to manual counting using the CellSearch system.
